# Inspiratory Muscle and Functional Performance of Patients Entering Cardiac Rehabilitation after Cardiac Valve Replacement

**DOI:** 10.3390/jcdd10040142

**Published:** 2023-03-28

**Authors:** Sabine Gempel, Meryl Cohen, Eryn Milian, Melany Vidret, Andrew Smith, Ian Jones, Yessenia Orozco, Neva Kirk-Sanchez, Lawrence P. Cahalin

**Affiliations:** 1 Department of Physical Therapy, University of Miami, Miller School of Medicine, Coral Gables, FL 33146, USA; 2 Department of Cardiac Rehabilitation, University of Miami Hospital, Miami, FL 33136, USA; 3 UF Health Cardiac & Pulmonary Rehab Gym, Gainesville, FL 32608, USA; 4 San Diego Gulls Hockey Club, Poway, CA 92064, USA; 5 Empowerme Wellness in Wickshire, Fort Lauderdale, FL 33309, USA

**Keywords:** valve replacement surgery, cardiac surgery, inspiratory muscle strength, maximal inspiratory pressure, sustained maximal inspiratory pressure, respiratory muscle performance, rehabilitation, cardiac rehabilitation

## Abstract

Limited research has examined inspiratory muscle performance (IMP) and functional performance (FP) of patients after valve replacement surgery (VRS). The purpose of this study was to examine IMP as well as several measures of FP in patients post-VRS. The study results of 27 patients revealed that patients undergoing transcatheter VRS were significantly (*p* = 0.01) older than patients undergoing minimally invasive or median sternotomy VRS with the median sternotomy VRS group performing significantly (*p* < 0.05) better than the transcatheter VRS group in the 6-min walk test, 5x sit-to-stand test, and sustained maximal inspiratory pressure. The 6-min walk test and IMP measures in all groups were significantly (*p* < 0.001) lower than predicted values. Significant (*p* < 0.05) relationships were found between IMP and FP with greater IMP being associated with greater FP. Pre-operative and early post-operative rehabilitation may improve IMP and FP post-VRS.

## 1. Introduction

Valvular heart disease is a commonly seen cardiac pathology that has historically been treated with invasive surgical interventions. However, research has shown that less invasive valve replacement surgery (VRS) results in reduced post-surgical complications and mortality rates [1,2]. The transcatheter (TC) approach has historically been used for high-risk patients and the median sternotomy (MS) has been utilized in younger healthier patients while the minimally invasive (MI) approach has been used in both high- and low-risk patients [3,4,5].

Regardless of which surgical technique is utilized, patients undergoing cardiac surgery have been found to have decreased respiratory muscle performance due to factors related to age, heart disease, and underlying comorbid conditions [6,7]. In fact, patients undergoing VRS demonstrate pre- and post-procedure respiratory muscle wasting worsening inspiratory muscle performance (IMP) [8]. While patients after VRS display some improvement in functional performance (FP) after 3 months, many measures of FP appear to remain below age- and gender-predicted values [9]. Alternatively, Cargnin et al. showed that 4 weeks of inspiratory muscle training (IMT) immediately following VRS was effective in restoring IMP to pre-operative levels and also increased FP assessed by the 6MWD [8].

The above findings demonstrate a need for pre- and post-operative cardiac rehabilitation, including IMT. A limited literature has examined IMP and FP in patients after VRS and few have examined the relationship between IMP and FP and to our knowledge, none have compared all three surgical approaches. The primary purpose of this study was to examine IMP as well as several measures of FP in patients undergoing MS-, TC-, and MI-VRS via a retrospective chart review with the primary outcome measures being baseline maximal inspiratory pressure (MIP), sustained maximal inspiratory pressure (SMIP), 6-minute walk test (6MWT), timed up and go (TUG), 5 time sit to stand (5xStS), Berg balance scale (BBS), and gait speed. The secondary purpose was to examine the relationship between IMP and FP following VRS. We hypothesized that the invasive nature of MS-VRS would result in poorer IMP and FP than after TC- and MI-VRS and that IMP and FP would be significantly correlated.

## 2. Materials and Methods

A retrospective chart review was performed on 27 patients’ post-VRS upon their entrance to the cardiac rehabilitation (CR) program at the University of Miami Health Systems between March and December 2019. To be included in this study, the patient had to be older than 18 years of age, undergone a VRS, and entered the CR program. The study was approved by the University of Miami Institutional Review Board (protocol # 20190543). Data was collected from the electronic medical record of each patient with demographic data including age, body mass index (BMI), sex, race and ethnicity, education level, health insurance status, comorbidities, occupation, type of VRS, and number of days since VRS. The primary outcome measures were taken during the initial evaluation in CR and included MIP, SMIP, 6MWT, TUG, 5xStS, BBS, Patient Health Questionnaire-9 (PHQ-9), chest wall expansion (CWE), and gait speed. The MI-VRS involved a right anterior thoracotomy with a 5–6 cm transverse incision in the right 2nd intercostal space [3] while the TC-VRS and MS-VRS were performed using standard procedures.

The MIP and SMIP were obtained via the test of incremental respiratory endurance (TIRE) protocol which was implemented via the PrO2 device (Design Net, Smithfield, USA) and was linked to a tablet. Subjects were standing with a nose clip and instructed to inspire forcefully after a maximal exhalation to residual volume (RV) and to continue inspiring for as long as possible to total lung capacity (TLC) [10]. Thus, both MIP and SMIP were obtained from RV with MIP measured at 1–2 s of inspiration and SMIP measured at TLC. The unit of measure for MIP and SMIP were centimeters of water (cm H_2_O) and pressure time units (PTU), respectively. The best MIP of three trials and the corresponding SMIP were then used for study purposes. Higher scores in both MIP and SMIP signify greater IMP with MIP reflecting inspiratory muscle strength and SMIP reflecting single-breath work capacity [11]. The following equations with height in cm were used to calculate the % of predicted values for (1) men: MIP = 152.0 − (age × 0.87) and SMIP = (height × 12.6) − (age × 9.95) − 1054.1 and (2) women: MIP = 107.2 − (age × 0.41) and SMIP = 630.9 − (age × 4.19) [11]. 

The 6MWT is a submaximal functional capacity and endurance assessment, with a score of <300 m signifying increased risk of death or poorer outcomes [12]. Gait speed was calculated by converting total distance walked in 6 min into meters per second. The TUG assesses fall risk, with a score of 13.5 s or greater indicating an increased risk of falls [13]. The 5xStS measures functional lower extremity strength and power with scores exceeding the following can be considered to have below average performance: 11.4 s (60–69 y/o), 12.6 s (70–79 y/o), 14.8 s (80–89 y/o) [14]. The BBS examines static and dynamic balance activities of varying difficulty, where scores of 40 or less may indicate an increased risk of falling [15].

Statistical analyses included descriptive statistics, the Kruskal–Wallis test with Bonferroni correction, Mann–Whitney U test, Fisher’s exact test, and Spearman Rho correlation analyses with the level of significance set at *p* < 0.05. All analyses were performed using IBM^®^ SPSS^®^ Statistics version 26. A priori sample size estimation was performed using G*Power 3.1.9.7 and data from previous publications focusing on known-groups’ validity and convergent validity of SMIP and its ability to distinguish between known-groups as well as correlation to functional performance, respectively. The Hedge’s g effect size of SMIP to distinguish between known-groups of airflow limitation was previously observed to be 0.671 (*p*-value = 0.003) [16] which ensured that a total sample size of 27, using ANOVA with *p* = 0.05 and 0.80 power (yielding an actual power of 0.84) for three groups was sufficient for this study.

## 3. Results

**Figure 1:**
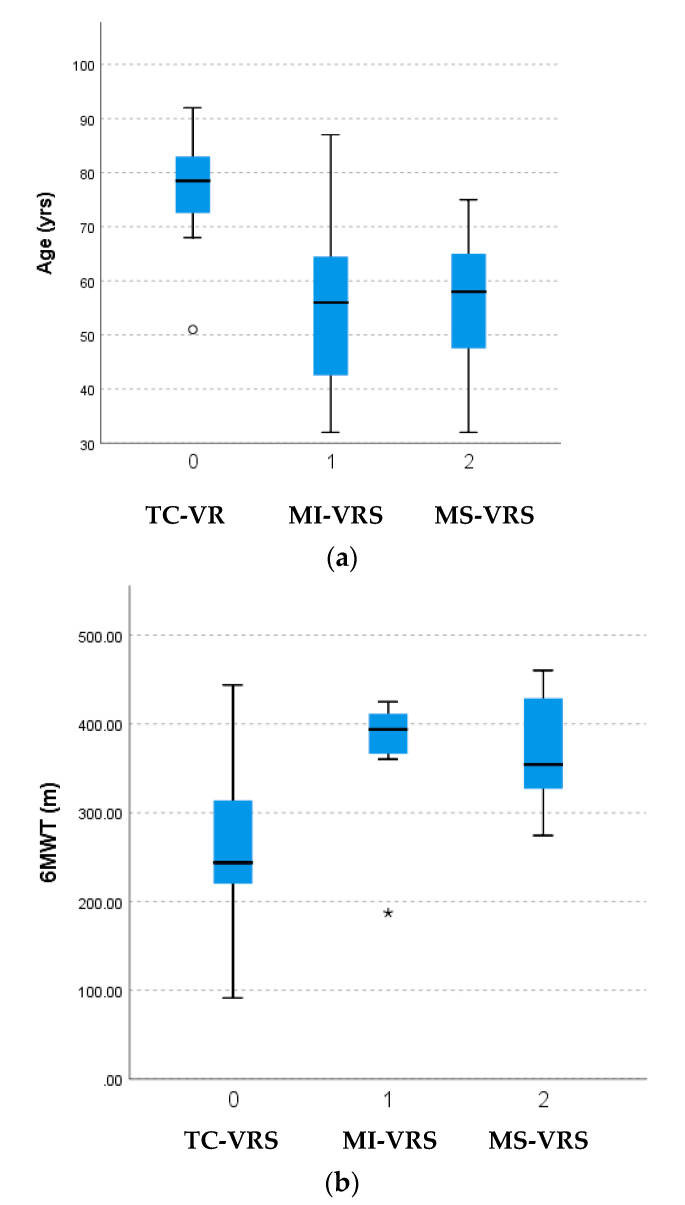

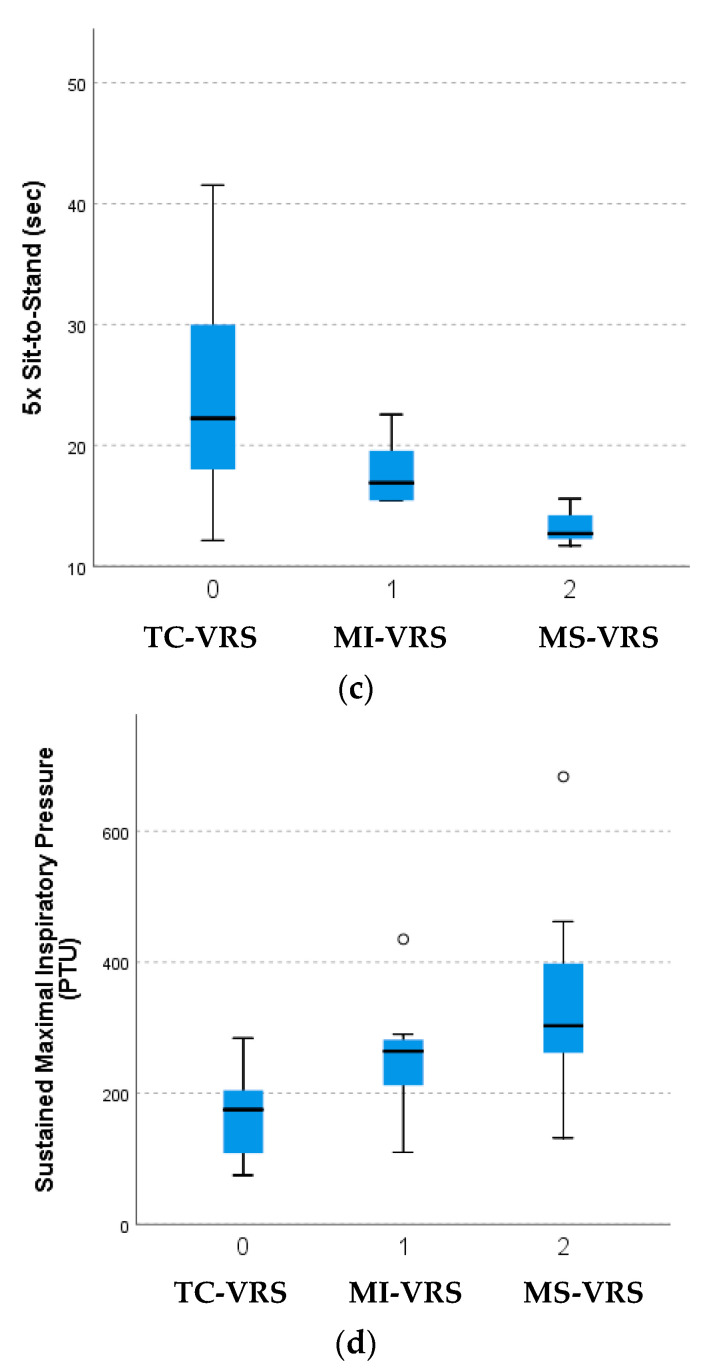
Key characteristics of valve replacement types. (**a**) Age of patients among the valve replacement types (VRS = valve replacement surgery; TC = transcatheter; MI = minimally invasive; MS = median sternotomy). (**b**) 6-min walk test performance of patients among the valve replacement types. (VR S= valve replacement surgery; TC = transcatheter; MI = minimally invasive; MS = median sternotomy). (**c**) 5x sit-to-stand performance of patients among the valve replacement types. (VRS = valve replacement surgery; TC = transcatheter; MI = minimally invasive; MS = median sternotomy). (**d**) Sustained maximal inspiratory pressure of patients among the valve replacement types. (VRS = valve replacement surgery; TC = transcatheter; MI = minimally invasive; MS = median sternotomy). An * in this figure on SPSS represents an extreme outlier.

The demographic and clinical characteristics of the patients are shown in Table 1 and Table 2. A total of 27 patients (11 males and 16 females) underwent VRS. The mean age of the 27 patients in the study was 65.0 (SD = 17.2) with a range of 32 to 92 years. The TC-VRS approach was the most common surgical approach (44%), followed by MI-VRS (30%) and MS-VRS (26%). Nineteen patients had aortic valve surgery (nine males and ten females), six patients had mitral valve surgery (two males and four females), and three female patients had tricuspid valve surgery, which included one patient who had both mitral and tricuspid valve surgery simultaneously. The type of valve disorder in each of the surgical approaches is shown in the legend of Table 1 and no significant difference in FP or IMP was found among types of valvular disorders (stenosis, regurgitation, bicuspid valve, and mass removal). No significant difference in demographic, clinical characteristics, or outcome measures were found between males and females. The major comorbidities of the subjects included hypertension (78%), hyperlipidemia (52%), diabetes (30%), coronary artery disease (30%), heart failure (22%), chronic obstructive pulmonary disease (19%), vascular disease (peripheral vascular disease, pulmonary embolism, cerebrovascular accident, and deep vein thrombosis;19%), aortic aneurysm (7%), and Marfan’s syndrome (7%) with no significant difference in the number of comorbidities among the surgical approaches. Additionally, the ejection fraction of the study subjects was not significantly different among groups regardless of surgical technique or type of valve disorder. The mean ± SD length of stay was 3.1 ± 2 days with a range of 1–8 days. Patients undergoing TC-VRS were significantly older than those undergoing MI-VRS or MS-VRS (Figure 1). While not statistically significant, the average time from surgery to entry into CR for all surgery types was 80.3 days, with the TC-VRS being the greatest followed by MS-VRS and MI-VRS (Table 1).

### 3.1. Inspiratory Muscle and Functional Performance

The MS-VRS group performed significantly better than the TC-VRS group in the 6MWT, 5xStS, and SMIP (Table 2). The MS-VRS group performed significantly better than the MI-VRS group in the 5xSTS while the MI-VRS group performed better than the TC-VRS group on the 6MWT (Figure 1). While no significant differences were found in % predicted 6MWT, MIP, and SMIP between groups, all groups had significantly (*p* < 0.0001) lower values than age and gender predicted values. No other significant differences were found among groups.

### 3.2. Correlation Analyses

**Figure 2:**
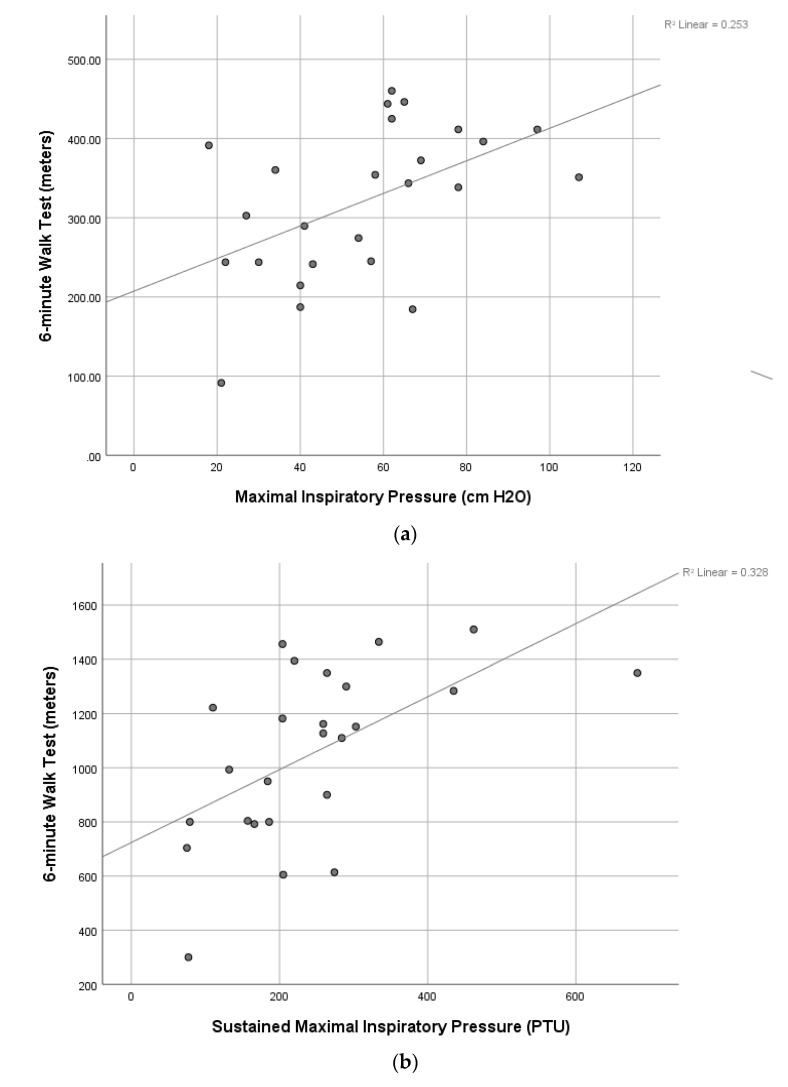
(**a**)**.** Scatterplot of the relationship between maximal inspiratory pressure and the 6-min walk test. (**b**). Scatterplot of the relationship between sustained maximal inspiratory pressure and the 6-min walk test.

**Figure 3:**
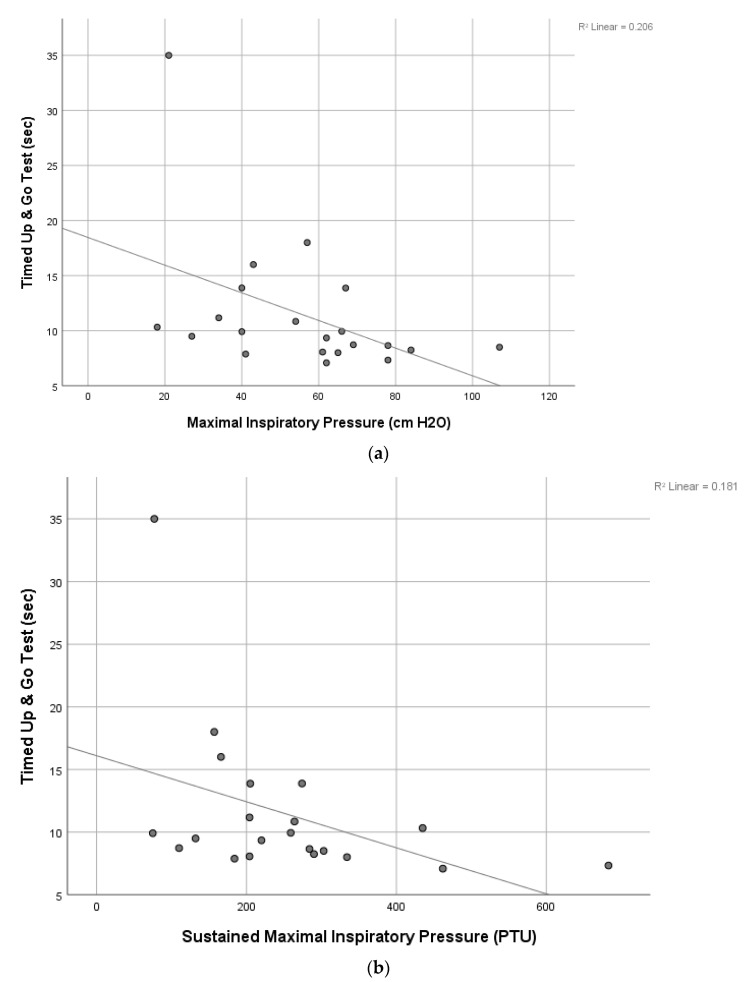
(**a**). Scatterplot of the relationship between maximal inspiratory pressure and the timed up and go test. (**b**). Scatterplot of the relationship between sustained maximal inspiratory pressure and the timed up and go test.

**Figure 4:**
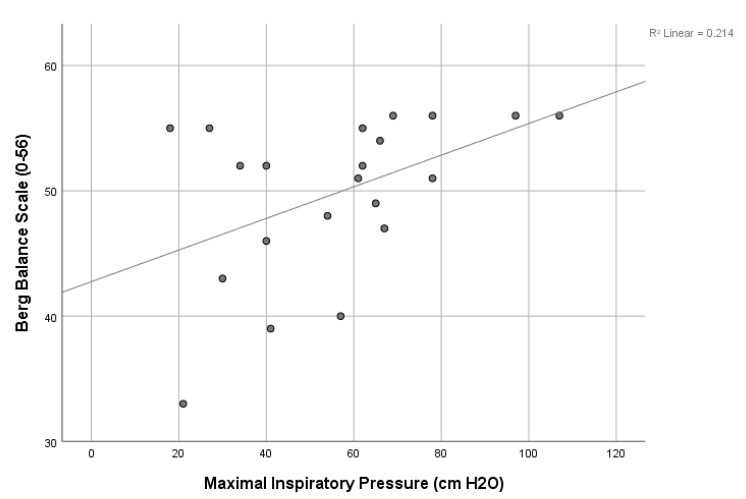
Scatterplot of the relationship between maximal inspiratory pressure and the Berg balance scale.

The results of correlation analyses found many significant relationships among IMP and FP (Table 3). Significant relationships (*p* < 0.05) were found between MIP and the SMIP, 6MWT, TUG, and BBS and between SMIP and the 6MWT and TUG. Age was significantly correlated negatively to all outcome measures (r-value range of −0.48 to −0.69; *p* < 0.05), except for a significant and near significant positive correlation to TUG (r = 0.52; *p* = 0.01) and 5xStS (r = 0.40; *p* = 0.06), respectively, indicating that FP worsened with increasing age. Scatterplots of several of the above significant correlations are shown in Figure 2, Figure 3 and Figure 4.

## 4. Discussion

The current study examined IMP and FP in patients undergoing three different VRS procedures with the hypothesis that the invasive nature of MS-VRS would result in poorer IMP and FP than after TC- and MI-VRS. We also hypothesized that IMP and FP would be significantly correlated, based on a limited number of studies observing such relationships. In our study, the TC patients were the oldest group with the greatest number of co-morbidities and had the poorest IMP and FP. This is consistent with research showing that TC-VRS is performed in higher risk patients who appear to have a poorer ability to tolerate or recover from surgery [4,17,18]. While our research did not include pre-operative assessment, a recent study by Hall et al. in 2022 in similarly aged patients undergoing similar surgical procedures, assessed respiratory muscle and functional performance measures both pre- and post-operatively and found statistically significant declines in MIP, 6MWT, and TUG following surgery [19]. Additionally, Kiomaki et al. in 2021 looked at functional performance via the 6MWT in patients undergoing SAVR vs. TAVR and divided both groups into “frail” or “non-frail via the Cardiovascular Health Study frailty index and examined them before and after surgery. A key finding was that frailty status did not impact 6MWT performance following surgery. They found that the SAVR group, regardless of frailty, declined significantly, whereas the TAVR group’s 6MWT decline did not reach statistical significance [20]. These results suggest that our subjects possibly had higher pre-operative measures as well. Most importantly, regardless of whether their functional and inspiratory muscle performance declined, remained similar, or improved after surgery, all three groups had functional and inspiratory muscle performance outcomes that were below predicted values.

Ribeiro et al. compared surgical aortic valve replacement (SAVR) and transcatheter aortic valve implantation (TAVI) patients and found TAVI patients were significantly older with more co-morbidities. Tarro-Genta et al. compared the same two VRS techniques and found TAVI patients had higher disability, co-morbidity profile, and risk of falls post-operatively, despite the groups being of similar age [21,22]. In contrast to our TC group, the MS patients were the youngest group with the lowest BMI and greatest IMP and FP. Additionally, in contrast to our results, Pardaens et al. found that patients after a MI mitral VRS had significantly better post-operative 6MWT distance than patients after a MS. However, the MS group was significantly older than the MI group, which is consistent with our findings highlighting that negative relationship between age and FP. The authors concluded that exercise capacity after cardiac surgery is related to preoperative risk and the type of surgery performed, which is also supported by our findings [23]. While our study did not show a statistically significant difference in average time (80.3 days) from surgery to admissions into CR among groups, TC-VRS was the greatest followed by MS-VRS and MI-VRS. This difference may be due to patients’ overall health status and/or different protocols based on VRS type or surgeon. This finding is clinically important because research has shown that for every day a patient delays starting cardiac rehabilitation there is a 1% decrease in the likelihood the patient will participate [24].

### 4.1. Effect of Surgical Technique on Inspiratory Muscle and Functional Performance

In our study we found a significant difference between surgical techniques in many measures of IMP and FP. However, the percent of predicted SMIP and 6MWT were not significantly different, highlighting the important role that age- and gender-predicted measures have in the assessment of IMP and FP. It is important to note that all patients, regardless of surgical technique, fell far below their predicted values with no VRS group above 70% of the predicted value. These results suggest impaired IMP and FP were likely present pre-operatively and worsened after the VRS. It is also noteworthy that while the 6MWT percent of predicted values were not statistically different among groups, the TC-VRS group was the only group to fall below 300 m which is associated with a poorer prognosis. Consistent with our results, Tarro-Genta et al. found that TC-VRS patients had significantly worse 6MWT, balance, and more co-morbidities post-operatively compared to MS-VRS of similar age [22]. Furthermore, while there was no statistical difference among the TUG scores of the three groups, the only group to score above 13.5 s was the TC-VRS group which is associated with an increased fall risk. Additionally, CWE was not significantly different among groups, but the only group to score within a normal range was the MI-VRS group. We hypothesize that the older TC group had diminished CWE due to age-related structural changes caused by a stiffening and calcification of the costal cartilage and costovertebral articulations [25], while the younger MS group experienced poorer CWE from the sternotomy and surgical manipulation. In our study, age was the only statistically significant demographic difference among the three groups, with the TC group representing the oldest population. The physiological effects of aging combined with valvular heart disease likely produced our observed findings of impaired IMP and FP rather than the VRS procedure. All of the above findings highlight the important role of pre-operative and post-operative efforts to improve IMP and FP which, based on the study by Cargnin et al., IMT alone may improve both IMP and FP [8].

### 4.2. Pulmonary and Inspiratory Muscle Impairments in Patients Undergoing Valve Replacement Surgery

Following cardiac valve surgery there is significant pulmonary dysfunction which is the primary cause of post-operative pulmonary complications (PPCs), morbidity and mortality [26]. This dysfunction includes impaired ventilation, reduced lung volumes, hypoventilation, and respiratory muscle impairments leading to dyspnea, ineffective cough, impaired gas exchange, increased work of breathing, increased inpatient length of stay, increased mechanical ventilation time, and impaired FP [26,27,28]. Thus, exercise and FP does not recover spontaneously after valve surgery and likely warrants CR. In fact, Westerdahl et al. showed a statistically significant decrease in pulmonary function up to one year after surgery and Hegazy et al. and Cargnin et al. both found a significant decrease in MIP following cardiac valve surgery [8,28]. Furthermore, Weber et al. found MIP to be a strong predictor of 90-day mortality and rehospitalization [29] with a reduction in respiratory muscle strength (<75% of predicted) associated with a higher incidence (almost six times greater relative risk) of PPCs. Although no pre-operative measures of IMP or FP were possible in our study, our results and the results of previous studies highlight the importance of pre-operative and post-operative examination and management of IMP and FP. Furthermore, early recovery of IMP via IMT has the potential to reduce the risk of PPCs since improvements in lung function, mechanical ventilation duration, length of stay, and FP have been found with IMT [26,28,29].

Previous research that did not differentiate between VRS techniques found impaired 6MWT, balance, 5xStS, pulmonary function via spirometry, MIP and MEP [8,9,26,28,30,31,32]. These studies examined IMP and FP either pre- or post-operatively and with or without intervention. Patients post-VRS have been found to significantly improve 6MWT, 5xStS, balance, and VO2 peak after CR. A systematic review and meta-analysis by Dsouza et al. found that the addition of IMT to usual care significantly reduced PPCs and LOS, and improved pulmonary function, 6MWT and MIP [26]. Studies examining the role of IMT in VRS includes both pre- and post-operative IMT. Ferreira et al. found that two weeks of IMT before VRS improved pulmonary function but not MIP [30]. Cordeiro et al. examined the effects of IMT after VRS to hospital discharge and found a significant improvement in 6MWT and MIP [31]. Cargnin et al. examined the effects of four weeks of IMT after VRS and found a significant improvement in MIP, 6MWT, and lung function [8]. Most recently, Hegazy et al. found that the addition of IMT to standard CR significantly improved pulmonary function and MIP to baseline values in 4 weeks, while patients not receiving IMT required 6 months to achieve baseline values [28]. Conversely, Saxena et al. and de Menezes et al. showed an improvement in a much shorter time frame in which pulmonary function returned to baseline values one month after VRS and MIP returned to baseline values just six days after VRS. However, neither Saxena et al. or de Menezes et al. examined FP nor did they comment on the provision of interventions during or after hospitalization [9,32]. The data in our study was collected when the participants were initially enrolled into CR and none of the patients received pre-operative testing or interventions, but all patients received standard post-operative inpatient care. Nonetheless, the variety and number of FP measures and the addition of the SMIP in our study is important and provides a comprehensive examination of FP and IMP in patient’s post-VRS.

### 4.3. Correlation of Inspiratory Muscle Performance to Functional Performance

The results of our correlation analyses found significant modest to good correlations between IMP and FP with the strongest positive correlation between SMIP and 6MWT and the strongest negative correlation between SMIP and age, highlighting the discriminatory ability of this novel measure of single-breath work capacity. The finding that both MIP and SMIP were significantly correlated to almost all FP measures is surprising. The MIP was significantly correlated to all FP measures except the 5xSTS while the SMIP was also not significantly correlated to the 5xSTS as well as the BBS. The finding that both MIP and SMIP were significantly correlated to an endurance measure like the 6MWT and a measure of functional mobility requiring static and dynamic balance like the TUG is important and provides insight into the dynamic role that the inspiratory muscles play in FP and balance. In fact, the MIP was also significantly correlated to the BBS, another common measure of static and dynamic balance, which suggests that inspiratory strength is important in maintaining balance in patients after VRS. Thus, IMP appears to impact many measures of FP which has been observed by others in patients post-VRS [8,21,32]. Cargnin et al., also found a significant relationship between MIP and 6MWT as well as pulmonary function and the 6MWT [8]. A systematic review and meta-analysis by Ribeiro et al. found a significant positive correlation between 6MWT and peak oxygen consumption after CR [21]. de Menezes et al. found a significant relationship between MEP and peripheral muscle strength (PMS). The MIP was not found to be correlated with PMS, which the authors hypothesized was due to diaphragm dysfunction after VRS [32]. The above findings provide support for the role IMP plays in FP and efforts to improve IMP via IMT and CR in patients undergoing VRS is likely to improve FP while also decreasing the risk of PPCs and length of stay. Although further research is needed to examine the effects of IMT on FP in patients undergoing VRS, the results of our study suggest that it may be warranted pre- and post-operatively.

The limitations of our study include: (1) a modest sample size, but similar to many studies examining FP and IMP associated with valve surgery and yielding an actual power of 0.84; (2) a lack of pre-operative FP and IMP data which is currently not standard practice at our institution and based on our findings appears to be important for healthcare providers to assess these measures pre-operatively; (3) an uneven group size which is consistent with the prevalence of valvular heart disease and valve surgery; and (4) a retrospective review. Despite the above limitations the detailed assessment of many FP and IMP outcomes makes the current study novel with potential to impact patient care pre- and post-operatively.

## 5. Conclusions

To the best of our knowledge, this is the first study to compare IMP and FP among three different VRS approaches (MI, TC, MS) in patients with aortic, mitral and tricuspid valvular disease. We also believe it is the first study to present data on single-breath work capacity measured by the SMIP, making our study relatively novel in this patient population. The results of our study found that regardless of surgical technique, all patients post VRS presented with poor inspiratory muscle strength (via MIP) and single-breath work capacity (via SMIP) that was far below age and gender-predicted values. This is consistent with previous research showing that patients requiring VRS present with impaired respiratory muscle strength both before and after surgery. The available literature has found that poor respiratory muscle performance is associated with greater PPCs including increased length of study, prolonged mechanical ventilation time, impaired gas exchange, reduced lung volumes, and greater morbidity and mortality. The results of our study and others suggest that IMT may be an important adjunct in the pre- and post-operative care provided to patients undergoing VRS. Further research on the effects of IMT methods such as dose and intensity as well as the effects of pre-operative versus post-operative IMT on outcomes in patients undergoing VRS appears warranted.

## Figures and Tables

**Table 1:** Demographic variables: mean (SD) or *n* (%).

Demographic Variable		All Patients (*n* = 27)	TC-VRS * (*n* = 12)	MI-VRS ** (*n* = 8)	MS-VRS *** (*n* = 7)	*p* Value
Age (years) ^†^		65.0 (17.2)	76.7 (10.3)	55.6 (17.4)	55.7 (15.4)	0.01 ^†^
Gender (male/female)		11 (40.7%)/16 (59.3%)	6 (50.0%)/6 (50.0%)	2 (22.2%)/7 (77.8%)	3 (50%)/3 (50%)	0.397
Ejection fraction (%)		48.57 (14.06)	48.00 (16.02)	50.00 (11.72)	48.33 (14.72)	0.889
Body mass index (kg/m^2^)		28.06 (5.60)	28.80 (6.77)	29.03 (3.57)	25.68 (5.31)	0.488
Occupation	Currently working	10 (40.0%)	4 (36.3%)	5 (55.6%)	1 (20.0%)	0.622
	Unemployed	3 (12.0%)	1 (9.1%)	1 (11.1%)	1 (20.0%)	
	Retired	11 (44.0%)	6 (54.5%)	2 (22.2%)	3 (60%)	
	Disability	1 (4%)	0 (0%)	1 (11.1%)	0 (0%)	
Race and ethnicity	African American	4 (14.8%)	0 (0%)	3 (33.3%)	1 (16.7%)	0.779
	Asian	2 (7.4%)	2 (16.7%)	0 (0%)	0 (0%)	
	White	20 (74.1%)	9 (81.8%)	6 (66.7%)	5 (83.3%)	
	Hispanic	18 (66.7%)	9 (81.8%)	5 (55.6%)	4 (66.6%)	
Education level	High school graduate	4 (28.6%)	1 (25.0%)	2 (28.6%)	1 (33.3%)	0.507
	2- or 4-year college	4 (28.6%)	1 (25.0%)	1 (14.3%)	2 (66.7)	
	Post-graduate	6 (42.9%)	2 (50.0%)	4 (57.1%)	0 (0%)	
Number of comorbidities		5.74 (3.07)	7.50 (2.93)	4.00 (2.13)	4.71 (2.87)	0.070
Days since surgery		80.29 (79.21)	70.64 (53.99)	43.57 (32.59)	140.83 (123.53)	0.070
Length of stay (# days) ^†^		3.14 (2.00)	1.91 (1.08)	4.75 (2.05)	4.00 (1.41)	0.006 ^†^
Exercised prior to surgery		11 (41%)	6 (50%)	4 (57.1%)	1 (17%)	0.267

* 9 aortic valve and 3 mitral valve procedures; ** 4 aortic valve, 1 mitral valve, 2 tricuspid valve, and 1 mitral and tricuspid valve procedure.; *** 6 aortic valve and 1 mitral valve procedure; ^†^ *p* < 0.05 via Kruskal–Wallis Test and Bonferroni correction.

**Table 2:** Outcome measures by type of surgery: mean (SD) or *n* (%).

	TC-VRS (*n* = 12)	MI-VRS (*n* = 8)	MS-VRS (*n* = 7)	*p* Values
Maximum inspiratory pressure (cmH_2_O) (MIP)	47.92 (18.35)	57.71 (28.38)	64.43 (24.34)	0.400
Sustained maximum inspiratory pressure (PTU) * (SMIP)	167.83 (67.99)	256.71 (99.10)	348.14 (177.52)	0.017 *
Chest wall expansion at xiphoid process (in) (CWE)	1.14 (0.64)	2.00 (0.71)	1.23 (0.80)	0.324
6-min walk test (m) * (6MWT)	258.76 (88.52)	369.39 (76.65)	371.44 (70.65)	0.035 *
5x sit to stand (s) * (5xSTS)	24.39 (9.03)	17.97 (3.06)	13.21 (1.59)	0.016 *
Timed up and go (s) (TUG)	13.77 (8.22)	10.29 (1.88)	8.54 (1.42)	0.116
Berg balance scale (BBS)	46.11 (7.81)	52.00 (4.12)	52.33 (3.45)	0.080
Patient health questionnaire-9 (PHQ-9)	7.88 (7.86)	6.33 (3.16)	10.20 (6.22)	0.427
% Predicted 6MWT	60.76 (19.27)	70.90 (9.74)	65.60 (15.55)	0.418
% Predicted MIP	58.77 (20.20)	66.09 (35.50)	67.30 (29.41)	0.759
% Predicted SMIP	57.44 (29.95)	61.88 (23.81)	60.67 (22.57)	0.262

* *p* < 0.05 via Kruskal–Wallis Test and Bonferroni correction.

**Table 3:** Spearman’s Rho correlation coefficients among key outcome measures.

	MIP (cmH_2_O)	SMIP (PTU)	6MWT (m)	Berg Balance Scale	TUG (s)	5x StS (s)	Age (yr)
MIP (cmH_2_O)	r = 1						
SMIP (PTU)	r = 0.505 *p* = 0.010 ^‡^	r = 1					
6MWT (m)	r = 0.476 *p* = 0.016 ^†^	r = 0.591 *p* = 0.002 ^‡^	r = 1				
Berg Balance Scale	r = 0.472 *p* = 0.031 ^†^	r = 0.310 *p* = 0.172	r = 0.565 *p* = 0.006 ^‡^	r = 1			
TUG (s)	r = −0.513 *p* = 0.017 ^‡^	r = −0.500 *p* = 0.021 ^†^	r = −0.761 *p* < 0.001 ^‡^	r = −0.443 *p* = 0.050	r = 1		
5x StS (s)	r = −0.284 *p* = 0.212	r = −0.292 *p* = 0.199	r = −0.635 *p* = 0.001 ^‡^	r = −0.439 *p* = 0.060	r = 0.716 *p* = 0.001 ^‡^	r = 1	
Age (years)	r = −0.555 *p* = 0.004 ^‡^	r = −0.661 *p* < 0.001 ^‡^	r = −0.688 *p* < 0.001 ^‡^	r = −0.683 *p* < 0.001 ^‡^	r = 0.517 *p* = 0.014 ^†^	r = 0.402 *p* = 0.064	r = 1

^†^ Spearman’s Rho correlation is significant at the 0.05 level (2-tailed); ^‡^ Spearman’s Rho correlation is significant at the 0.01 level (2-tailed); MIP = maximum inspiratory pressure, SMIP = sustained maximum inspiratory pressure; 6MWT = 6 min walk test; TUG = timed up and go; 5x StS = 5 time sit-to-stand.

## Data Availability

The data can be made available upon request to the corresponding author.
